# Supplementary material for: Venomix: a simple bioinformatic pipeline for identifying and characterizing toxin gene candidates from transcriptomic data
Source: PeerJ. 2018 Jul 31;6:e5361. doi: 10.7717/peerj.5361 (PMC6074769; doi:10.7717/peerj.5361)
Supplement: Supplemental Information 3 [file peerj-06-5361-s003.gz › FinalOutput_E-20/Kunitz-type_serine_protease_inhibitor_bitisilin-3_1/finaltree.pdf]

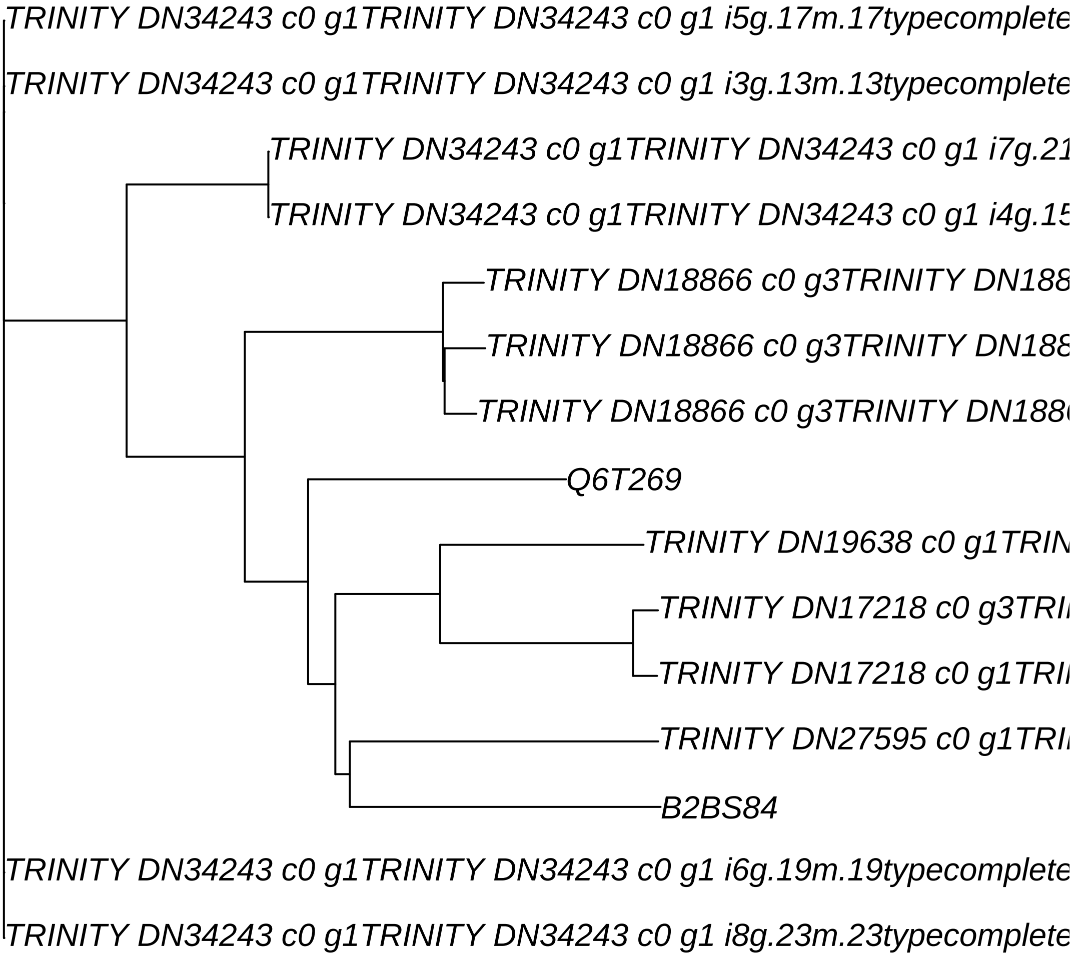
TRINITY DN34243 c0 g1TRINITY DN34243 c0 g1 i5g.17m.17typecomplete
TRINITY DN34243 c0 g1TRINITY DN34243 c0 g1 i3g.13m.13typecomplete
TRINITY DN34243 c0 g1TRINITY DN34243 c0 g1 i7g.21
TRINITY DN34243 c0 g1TRINITY DN34243 c0 g1 i4g.15
TRINITY DN18866 c0 g3TRINITY DN188
TRINITY DN18866 c0 g3TRINITY DN188
TRINITY DN18866 c0 g3TRINITY DN188
Q6T269
TRINITY DN19638 c0 g1TRIN
TRINITY DN17218 c0 g3TRI
TRINITY DN17218 c0 g1TRI
TRINITY DN27595 c0 g1TRI
B2BS84
TRINITY DN34243 c0 g1TRINITY DN34243 c0 g1 i6g.19m.19typecomplete
TRINITY DN34243 c0 g1TRINITY DN34243 c0 g1 i8g.23m.23typecomplete
